# Asthma exacerbation prevalence during the COVID-19 lockdown in a moderate-severe asthma cohort

**DOI:** 10.1136/bmjresp-2020-000758

**Published:** 2021-05-05

**Authors:** Geertje de Boer, Gert-Jan Braunstahl, Rudi Hendriks, Gerdien Tramper-Stranders

**Affiliations:** 1 Pulmonary Medicine, Franciscus Gasthuis and Vlietland, Rotterdam, The Netherlands; 2 Pulmonary Medicine, Erasmus MC, Rotterdam, The Netherlands; 3 Pediatrics, Franciscus Gasthuis and Vlietland, Rotterdam, The Netherlands

**Keywords:** asthma, viral infection, infection control

## Abstract

**Introduction:**

Following the recent COVID-19 lockdown, a reduction in emergency healthcare visits was reported. Infectious diseases were less often diagnosed, while it was not clear if this was due to a decrease in prevalence or a decrease in emergency healthcare visits due to fear of COVID-19.

**Methods:**

This study comprises a follow-up from a recently finished randomised controlled trial, to gain insight into the prevalence of asthma exacerbation and fear of COVID-19 in patients with moderate-severe asthma and controls in the Netherlands. Participants, patients with asthma and controls, were invited to fill out a short survey by email or post. Exacerbation frequencies until 1 July 2020 were verified with the hospitals’ and general practitioners’ medical records, pharmacies and patient interviews.

**Results:**

In quarter 2 of 2020, mean exacerbation frequency per patient was significantly lower (χ^2^(3)=9.91, p=0.019) compared with quarter 2 in previous years. Patients with asthma were more likely to avoid (38.8%; controls, 0.0%, p<0.01) or delay (24.5%; controls, 0.0%, p=0.02) essential medical visits due to fear of SARS-CoV-2 infection at medical facilities.

**Conclusion:**

In conclusion, we found a significantly reduced asthma exacerbation frequency during COVID-19 social distancing measures compared with previous years. Patients with asthma also showed more anxiety towards (acquiring) COVID-19 infection.

**Trial registration number:**

NL8576.

**Key messages**

- A decrease in asthma exacerbation and an increase of fear towards COVID-19 were measured among patients with asthma.
- While patients with asthma experience less asthma exacerbation during COVID-19 lockdown, they also seem to experience more fear towards COVID-19 compared with controls without respiratory disease.

## Introduction

Following the recent COVID-19 lockdown, a reduction in emergency healthcare visits was reported.^1^ Infectious diseases were less often diagnosed, probably due to social distancing and increased hygiene measures. However, also fear of acquiring COVID-19 infection at medical facilities might have led to a decrease in visits.^1 2^

Recent studies have indicated that controlled allergic asthma is not a risk factor per se for severe disease in the current COVID-19 pandemic.^2–5^ Entry of the SARS-CoV-2 virus is mediated by interferon-driven upregulation of ACE2. In patients with asthma with type 2 inflammation, airway interferon responses are known to be deficient.^6^ This might confer protection against severe COVID-19 symptoms.^3 4 6 7^

Social distancing is known to reduce the spread of seasonal influenza and viral respiratory tract infections (RTIs).^2 8^ We hypothesise that this reduction in viral transmission during the COVID-19 lockdown is responsible for a decrease in asthma exacerbations (AEs), which is often elicited by viral RTI. Therefore, in a cohort of patients with moderate-severe asthma and recurrent AEs, we analysed the effect of the COVID-19 lockdown on (1) the frequency of severe AEs requiring oral corticosteroids and (2) healthcare avoidance.

## Methods

This study is registered in the Dutch trial register. Patients with asthma who recently completed follow-up from the Breathe Study (NL5752), a clinical trial in which patients were randomised to bacterial lysate and placebo therapy and followed for 24 months, were included. Primary outcome in this study was the number of exacerbation within 18 months of study enrolment. Controls without asthma that recently completed a cross-sectional study, the Grandma Study (NCT03278561), were included for comparison. Primary outcomes for this study were immunological parameters in adult onset asthma.

All patients with asthma were diagnosed according to the Global Initiative for Asthma (GINA) guidelines. Patients with asthma were treated with at least GINA step 3 medication: medium/high-dose inhaled corticosteroids and long-acting beta2-agonists. Controls were free from pulmonary diseases, but not per se from other comorbidities.

### Patient and public involvement

A selection of participants was involved in the conduct of this research. They advised on questions in the COVID-19 survey and the included questionnaires. All participants will be informed of the results through a dedicated website on the randomised controlled trial they already participated in (www.breathestudie.nl). Also, the results will be sent by email as a study newsletter, suitable for a non-specialist audience.

Participants were invited to fill out a short survey by email or post (see online supplemental material), including the Asthma Control Questionnaire (ACQ), the Asthma Quality of Life Questionnaire (AQLQ) and the Hospital Anxiety and Depression Scale (HADS), as well as questions concerning exacerbation frequency and care avoidance between 12 April and 1 June 2020.^9 10^ Two reminders were sent out. Exacerbation frequencies until 1 July were verified with the hospitals and general practitioners’ medical records, pharmacies and patient questionnaire. In case of inconsistencies, patients were interviewed. COVID-19 restrictions in the Netherlands started mid-March and were being lifted in a stepwise fashion as of early June 2020.

10.1136/bmjresp-2020-000758.supp1 Supplementary data

Clinical parameters from the original study visits were used for baseline characteristics. Primary outcome was the difference in number of AEs between the second quarter (Q2) of 2020 and the 3 years prior to the COVID-19 pandemic. Only severe AEs needing oral corticosteroids and/or antibiotics were included. Secondary outcomes were ACQ, AQLQ, HADS scores, healthcare avoidance and fear of COVID-19. ACQ and AQLQ were compared with April 2019.

Differences between asthma and controls were evaluated with the Χ^2^ test, Student’s t-test or the Mann-Whitney U test, depending on the variable and its distribution. Differences between time points were compared with a Friedman test. Bonferroni correction was applied for the Wilcoxon signed-rank test where a p value was set at 0.0083. Data are shown in median (25th–75th) or mean values±SD. Statistical analyses were conducted with SPSS V.26.0.

## Results

Out of 94 invited participants (67 asthma; 27 controls), 67 participants (~71%) provided informed consent and were included. Patients with asthma were mainly patients with severe and uncontrolled asthma, treated according to GINA 4. No significant differences were observed in age, body mass index, comorbidities and other demographics such as prevalence of proven COVID-19 (table 1).

**Table 1:** Participant demographics

	Controls N=18	Asthma N=49	P value
Female gender	10 (55.6)	39 (79.6)	0.06
Age	47.00 (30.75–62.50)	46.00 (32.50–54.50)	0.24
Body mass index	28.16±4.08	27.67±4.08	0.77
Asthma Control Questionnaire T0		2.00 (1.17–2.50)	.
GINA 3/GINA 4, N		12/37	.
Allergic sensibilisation	8 (44.4)	32 (65.3)	0.16
Smoking Never/quit	15/3	40/3	1.00
Annual influenza vaccination	9 (50.0)	50 (64.1)	0.27
Household composition with children <12 years	5 (29.4)	2 (6.5%)	0.08
Comorbidities	10 (55.56)	3 (6.12)	0.58
COVID-19 infected Yes/no/probable	1/16/1	0/43/6	0.65
COVID-19-infected family Yes/no/probable	1/16/1	5/41/3	0.89
Fear of acquiring COVID-19	2.50 (1.00–6.25)	5.00 (3.00–7.00)	0.04
Fear of acquiring COVID-19 at medical facility (yes)	0 (0.0)	22 (44.9)	<0.01
Essential medical visits:			
Avoidance	0 (0.0)	19 (38.8)	<0.01
Delayed	0 (0.0)	12 (24.5)	0.02

Data shown in mean±SD and median (25th–75th) or absolute counts N (%).

GINA, Global Initiative for Asthma.

**Figure 1:**
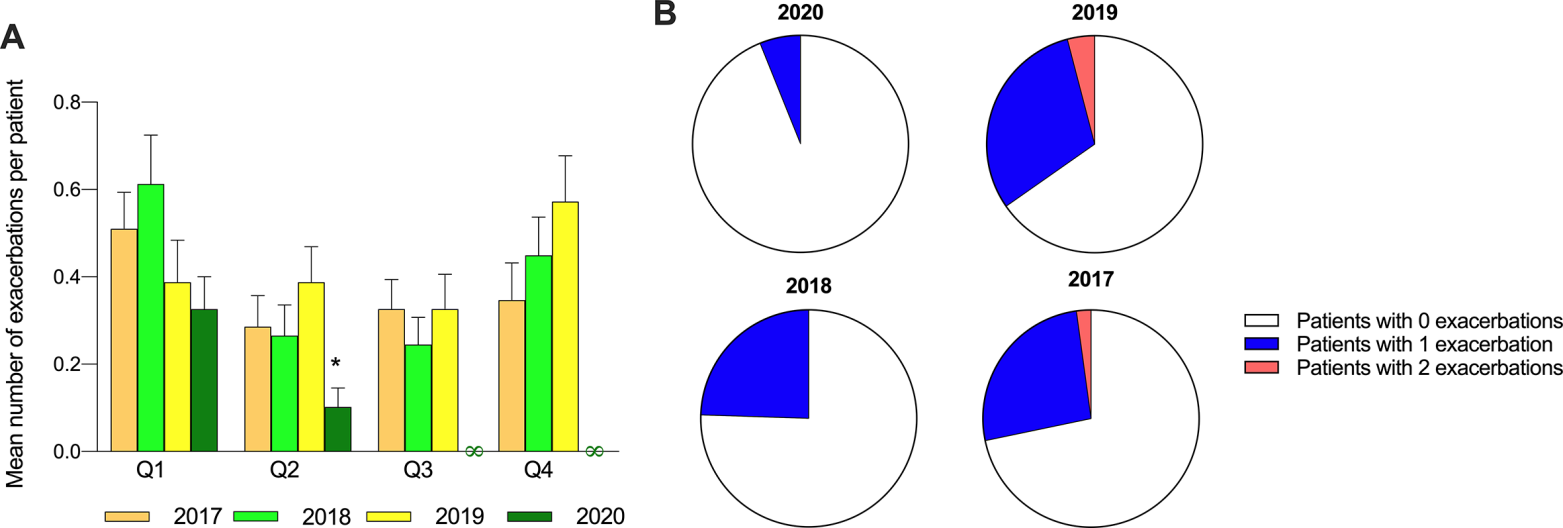
Asthma exacerbation in the years 2017–2020. (A) Mean number of exacerbation per patient per quarter per year; *statistically significant from Q2 in other years, ∞undefined. (B) Number of patients with 0, 1, 2 exacerbation frequencies in Q2 per year.

In Q2 of 2020, mean exacerbation frequency per patient was significantly lower (χ^2^(3)=9.91, p=0.019), compared with Q2 of 2017 (Z=−2.67, p=0.008), 2018 (Z=−2.50, p=0.012) and 2019 (Z=−3.26, p=0.001). No difference in mean exacerbation frequency was seen between Q1 of 2017, 2018, 2019 and 2020. Mean exacerbation frequency per patient per quarter did not differ between the years 2017, 2018 and 2019 (figure 1). AEs in 2020 were not related to positive SARS-CoV-2 PCR or hospitalisations. Asthma control and AQLQ in Q2 of 2020 were comparable with April 2019.

Patients with asthma were more likely to avoid (38.8%; controls, 0.0%, p<0.01) or delay (24.5%; controls, 0.0%, p=0.02) essential medical visits due to fear of SARS-CoV-2 infection at medical facilities. In case of AE, most patients used e-consults (83.3%). Fear was objectified by a clinically relevant higher HADS score reflecting a possible anxiety or depression disorder in patients with asthma compared with controls (8.00 (5.00–12.50) vs 4.00 (1.00–7.00), p<0.01).

## Discussion

In this study, we identified a decrease in AE during the COVID-19 lockdown measures in patients with moderate-severe asthma. We expect social distancing and fear towards acquiring COVID-19 to be responsible for this decrease.

Our original study cohort was designed to investigate the effect of bacterial lysates on AE frequency. Patients used either a bacterial lysate or placebo during the winter seasons of 2017/2018 or 2018/2019. Since the COVID-19 lockdown started almost a year later, and both patients with bacterial lysate and placebo use participated in the current study, we do not expect to find an effect of bacterial lysates on AE frequency.

Although the sample size in our study was small, it is of note that frequencies of AEs during the COVID-19 pandemic could be compared with baseline data acquired by quarterly monitoring of AEs over the past 3 years. Exacerbation frequencies in 2018 and 2017 were indeed higher compared with 2019 and 2020. Nevertheless, IQRs were large and it is possible that there was regression towards the mean. Exacerbation frequency in Q2 for 2020 is significantly lower compared with Q1 in 2020 and all other quarters in previous years. However, it might be possible that AEs were missed. Although we counterchecked AE frequency using different resources, there is still a possibility that patients did not report their AE anywhere. Also, we do not have information of patients who did not respond to the surveys.

Nevertheless, social distancing is known to reduce RTIs in children and influenza in adults.^2 8^ Therefore, we suggest abiding the COVID-19 lockdown is responsible for AE reduction. Q2 is a known period for aeroallergens and RTIs to increase AE prevalence in the Netherlands. RTIs might be reduced by social distancing. The COVID-19 lockdown also could have reduced aeroallergen exposure, as people mainly worked from their homes and outdoor exposition was decreased. Therefore, AE prevalence could have benefited from both, social distancing and reduced environmental exposures.

Patients with asthma experienced more fear of SARS-CoV-2 infection compared with controls. In a different study in which patients with asthma (GINA 3–4) were compared with controls without respiratory disease, we found no differences in HADS scores at baseline.^11^ Follow-up data showed comparable results with the current study, with HADS scores during COVID-19 lockdown increasing only in patients with asthma and not in controls.^12^ Nevertheless, fear of acquiring a SARS-CoV-2 infection at medical facilities did not lead to missed AEs or seriously delayed care for an AE because of e-consulting possibilities. Also, no patient with an AE was seen at the emergency department. It is possible that fear of acquiring COVID-19 in patients with asthma reduces the COVID-19 infection rate in patients with asthma in the Netherlands.

Conflicting reports on COVID-19 risks and asthma might confuse patients with asthma and increase their fear of acquiring COVID-19. Recent studies indicate that allergic asthma is not a risk factor for severe disease in the current COVID-19 pandemic and that type 2 inflammation might be protective.^2–5^ Thus, an increased fear of COVID-19 experienced by patients with allergic asthma seems irrational. However, asthma was described as a top three comorbidity in younger patients who were hospitalised for COVID-19, next to obesity and diabetes, in the USA.^13^ Possibly, asthma heterogeneity is responsible for the different outcomes with some evidence for type 2 high asthma not conferring a higher risk. We suggest information on COVID-19 disease should be adequate and more patient specific.

While patients with asthma would rather delay or avoid visits to medical care facilities, they did reach out via e-consults if needed. Our study suggests usage of an e-consult underlines the importance of a good communication platform between patients and their healthcare provider.

In conclusion, we found a significantly reduced AE frequency during COVID-19 social distancing measures compared with previous years. Patients with asthma also showed more anxiety towards (acquiring) COVID-19 infection. Because the risk of acquiring COVID-19 infection will be present for a yet unknown period, it is important to encourage them to contact their practitioner by e-consults.

## Data Availability

Data are available upon reasonable request, within 12 months after publication. After 12 months, requests can still be done, however, help from the authors cannot be guaranteed.
