# Supplementary material for: Asthma exacerbation prevalence during the COVID-19 lockdown in a moderate-severe asthma cohort
Source: BMJ Open Respir Res. 2021 May 5;8(1):e000758. doi: 10.1136/bmjresp-2020-000758 (PMC8102860; doi:10.1136/bmjresp-2020-000758)
Supplement: Supplementary data [file bmjresp-2020-000758supp001.pdf]

Online supplementary material: Asthma exacerbation prevalence during the Covid-19COVID-19 lockdown in a moderate-severe asthma cohort

G.M. de Boer et al.

Questionnaire

Date:

Study number:

Are you diagnosed with asthma by a doctor?
Yes / No

Have you been vaccinated for seasonal flu for the season 2019/2020?
Yes / No

What is your household size? ____

What medications do you take?

____
____
____

How often did you experience a respiratory tract infection in 2020? _______times

How often did you experience a period of fever from March 2020 onwards?
_______times

If present, was this fever present during a respiratory tract infection? Yes / No

How many days have you been stayed at home because of a respiratory tract infection or fever?

Respiratory tract infection: _______ days from March 2020 onwards

Fever: _____ days from March 2020 onwards

How many asthma exacerbations did you experience for which antibiotics and/or prednisone were prescribed?

- Between April 2019 and October 2019: _____
- Between October 2019 and March 2020: _____
- From March 2020 onwards: _____

Did you experience COVID-19 disease? Yes / No / Unsure

Did anyone in your household or close family experience COVID-19? Yes / No / Unsure

On a 1-10 scale: are you worried to acquire COVID-19? (1=not at all – 10=very much)

1 2 3 4 5 6 7 8 9 10

On a 1-10 scale: are you worried to acquire an asthma exacerbation? (1=not at all – 10=very much)

1 2 3 4 5 6 7 8 9 10

Did you avoid your general practitioner, the hospital emergency department or other essential medical care due to Covid-19? Yes / No

Did you delay essential medical care due to Covid-19? Yes / No

Are you afraid of acquiring COVID-19 when you visit your general practitioner or the hospital?
Yes / No
